# Prostatic cancers: understanding their molecular pathology and the 2016 WHO classification

**DOI:** 10.18632/oncotarget.24515

**Published:** 2018-02-16

**Authors:** Kentaro Inamura

**Affiliations:** ^1^ Division of Pathology, The Cancer Institute; Department of Pathology, The Cancer Institute Hospital, Japanese Foundation for Cancer Research, 3-8-31 Ariake, Koto-ku, Tokyo 135-8550, Japan

**Keywords:** genetic alteration, histology, molecular pathological epidemiology, prostate cancer, The Cancer Genome Atlas (TCGA)

## Abstract

Accumulating evidence suggests that prostatic cancers represent a group of histologically and molecularly heterogeneous diseases with variable clinical courses. In accordance with the increased knowledge of their clinicopathologies and genetics, the World Health Organization (WHO) classification of prostatic cancers has been revised. Additionally, recent data on their comprehensive molecular characterization have increased our understanding of the genomic basis of prostatic cancers and enabled us to classify them into subtypes with distinct molecular pathologies and clinical features. Our increased understanding of the molecular pathologies of prostatic cancers has permitted their evolution from a poorly understood, heterogeneous group of diseases with variable clinical courses to characteristic molecular subtypes that allow the implementation of personalized therapies and better patient management. This review provides perspectives on the new 2016 WHO classification of prostatic cancers as well as recent knowledge of their molecular pathologies. The WHO classification of prostatic cancers will require additional revisions to allow for reliable and clinically meaningful cancer diagnoses as a better understanding of their molecular characteristics is obtained.

## INTRODUCTION

Prostatic cancers possess substantial heterogeneity in their molecular alterations and variable clinical courses [1–43]. An increased understanding of their morphologies, immunohistochemistries, and associations with clinical features has mandated the World Health Organization (WHO) to revise its classification of prostatic cancers. Additionally, emerging evidence suggests that prostatic cancers can be classified into various subtypes, which have distinct molecular pathologies and clinical features. This review introduces and briefly summarizes the clinicopathologically important issues of the new 2016 WHO classification of prostatic cancers [1] as well as presents a new understanding of their molecular characteristics.

## THE 2016 WHO CLASSIFICATION

The 2004 WHO classification of prostatic cancers [44] precedes the current 2016 classification [1] that reflects the remarkable gains in our knowledge of the pathologies and genetics of prostatic cancers acquired during the intervening 12 years. The main differences between the previous and new WHO classifications are as follows: i) The Gleason grading system is modified to more accurately represent clinical outcomes. ii) Intraductal carcinoma of the prostate (IDC-P) and large cell neuroendocrine carcinoma (LCNEC) are newly recognized as subtypes of prostatic carcinoma. iii) The histological variants of acinar adenocarcinoma are updated. iv) New immunohistochemical markers are described, which are useful for diagnosis. Perspectives on the important issues addressed in the revised 2016 WHO classification are described below.

### Grading of prostatic adenocarcinoma

**Figure 1:**
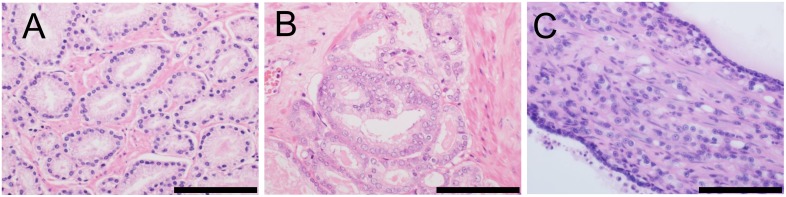
Morphology of distinct Gleason patterns (GPs) (**A**) Individual, discrete, well-formed glands (GP 3). (**B**) Fused/cribriform glands (GP 4). (**C**) Individual neoplastic cells infiltrating the stroma between benign glands (GP 5). Scale bar, 100 μm.

Gleason grading, the standard approach for assigning a histological grade to prostatic adenocarcinomas (Figure 1), is modified in the 2016 WHO classification [1, 4] according to discussions at the meeting of the International Society of Urological Pathology (ISUP) in 2014 [45]. The modifications are as follows: i) Cribriform glands and glomeruloid glands should be assigned Gleason pattern (GP) 4, regardless of morphology. ii) The GP of mucinous adenocarcinoma should be determined according to its underlying growth pattern. iii) IDC-P should not be assigned a GP, and a comment about its association with aggressive prostatic cancer should be provided. iv) GP 4 should include cribriform, fused, and poorly formed glands. v) GP 4 should be diagnosed under 10 × magnification. vi) Occasional/seemingly poorly formed glands between well-formed glands should be assigned GP 3. vii) In cases with a borderline morphology between GP 3 and GP 4, with crush artifacts, GP 3 should be favored. viii) Solid medium-to-large nests with a rosette-like structure should be assigned GP 5. ix) The presence of unequivocal comedonecrosis, even if focally observed, should be assigned GP 5. x) When the highest GS is 3 + 4 = 7 or 4 + 3 = 7, the percentage of GP 4 tumors should be reported [1, 45].

A new grading system specified by the 2016 WHO classification is supported by a broad consensus reached at the 2014 ISUP conference [45]. The histological definitions within this new grading system are provided in Table 1. The basis of this new grading system was proposed in 2013 [46] and was subsequently validated by a multi-institutional study [47]. The 5-year biochemical risk-free survival rates for the grade groups 1–5 are 96%, 88%, 63%, 48%, and 26%, respectively [47]. This new grading system can be used to predict mortality risk after radical prostatectomy for patients with grade groups 4 and 5. Prostatic cancer-specific survival is significantly shorter for patients with grade group 5 than patients with grade group 4 for the biopsy (hazard ratio [HR] = 2.13, 95% confidence interval [CI] = 1.37–3.30) and radical prostatectomy groups (HR = 2.38, 95% CI = 1.74–3.28) [48].

**Table 1:** Histological definitions of the new grading system (Grade Groups) [1]

Grade Groups	Histological Definitions
Grade Group 1	Gleason score ≤ 6 Only individual, discrete, well-formed glands
Grade Group 2	Gleason score 3 + 4 = 7 Predominantly well-formed glands with a few poorly-formed/fused/cribriform glands
Grade Group 3	Gleason score 4 + 3 = 7 Predominantly poorly-formed/fused/cribriform glands with a few well-formed glands
Grade Group 4	Gleason score 4 + 4 = 8; 3 + 5 = 8; 5 + 3 = 8 Only poorly-formed/fused/cribriform glands; Predominantly well-formed glands with a few areas lacking glands (or with comedonecrosis); Predominantly lacking glands (or with comedonecrosis) and a few well-formed glands
Grade Group 5	Gleason score 9–10 Lacking gland formation (or with comedonecrosis) with/without poorly-formed/fused/cribriform glands

### Subtypes of prostatic carcinoma

The new 2016 WHO classification updates the subtypes of prostatic carcinoma, as shown in Table 2. The newly adopted IDC-P and modification of neuroendocrine carcinoma (see below) are novel changes, acquired through updated knowledge of their biology and clinical usefulness. The histopathological and molecular characteristics of each subtype are discussed below, except for acinar adenocarcinoma. The latter comprises most prostatic cancers and is described according to histopathology and genomics in other sections.

**Table 2:** The 2016 and 2004 WHO classifications of prostatic carcinoma [1, 44]

2016 WHO classification	2004 WHO classification
Glandular neoplasms	Glandular neoplasms
Acinar adenocarcinoma	Acinar adenocarcinoma
Intraductal carcinoma	Ductal adenocarcinoma
Ductal adenocarcinoma	
Urothelial carcinoma	Urothelial carcinoma
Squamous neoplasms	Squamous neoplasms
Adenosquamous carcinoma	Adenosquamous carcinoma
Squamous cell carcinoma	Squamous cell carcinoma
Basal cell carcinoma	Basal cell carcinoma
Neuroendocrine tumors	Neuroendocrine tumors
Adenocarcinoma with neuroendocrine differentiation	Endocrine differentiation within adenocarcinoma
Small cell neuroendocrine carcinoma	Small cell carcinoma
Large cell neuroendocrine carcinoma	

WHO, World Health Organization.

### IDC-P

The 2016 WHO classification includes IDC-P as a new subtype of prostatic cancer [1, 4]. The definition of IDC-P is an “intra-acinar and/or intraductal neoplastic epithelial proliferation that has some features of high-grade prostatic intraepithelial neoplasia (HGPIN) but exhibits much greater architectural and/or cytological atypia, typically associated with high-grade, high-stage prostate carcinoma” [1, 4]. IDC-P manifests as the intraductal spread of advanced prostatic carcinoma, but may also reflect a preinvasive carcinoma derived from HGPIN [49, 50]. Isolated IDC-P without concomitant prostate carcinoma is detected in only 0.26% of prostate biopsies [51].

Genetically, in radical prostatectomy specimens, loss of heterozygosity (LOH) is detected in 29% and 60% in GP 4 carcinomas and IDC-Ps, respectively, whereas LOH is rarely observed in GP 3 carcinomas or HGPINs [52]. Although *ERG* rearrangement is less common in HGPINs, it is frequently observed (75%) in IDC-Ps [1, 53]. Loss of immunohistochemical detection of cytoplasmic PTEN is frequently observed (84%) in IDC-Ps, but rarely in HGPINs [1, 54]. Results from the above-mentioned genetic and immunohistochemical studies support the view that IDC-P represents a lesion distinct from HGPIN and a late event in prostate carcinogenesis [1, 4].

### Ductal adenocarcinoma

Ductal adenocarcinoma is the second most common (3%) subtype of prostatic carcinoma, which is better characterized clinically, histologically, and molecularly than other subtypes. Mixed ductal–acinar adenocarcinoma is more common than pure ductal adenocarcinoma, which accounts for only 0.2%–0.4% of prostatic carcinomas [1]. Ductal adenocarcinomas are frequently located in the periurethral area. Periurethral ductal adenocarcinomas often protrude into the urethra; hematuria and other urinary tract symptoms are common manifestations. The clinical findings for peripheral ductal adenocarcinomas are similar to those of acinar adenocarcinomas. For example, most patients have elevated levels of serum prostate-specific antigen (PSA); however, the levels are lower than those in patients with acinar adenocarcinoma. Clinical stage is often more advanced than that of typical acinar adenocarcinoma. In a population-based study, 12% of patients with ductal adenocarcinoma presented with distant metastasis compared with 4% of patients with acinar adenocarcinoma [55]. Ductal adenocarcinoma may metastasize to the penis, testis, and lung [1, 2, 56].

Ductal adenocarcinoma often presents as an exophytic, villous/polypoid mass arising from the verumontanum. The macroscopic appearance of peripheral ductal adenocarcinoma is similar to that of acinar adenocarcinoma [1, 2]. Ductal adenocarcinomas form ducts/acini with cribriform or papillary growth of tall columnar cells with elongated, frequently pseudostratified nuclei and an amphophilic, but occasionally clear, cytoplasm. Typical observations include large nucleoli, coarse chromatin, mitotic figures, and intraluminal necrotic debris. Papillary infoldings have fibrovascular cores, and cribriform spaces are typically slit-like. GP is usually 4 and is less frequently assigned GP 3 (PIN-like pattern) or GP 5 (solid or comedo pattern).

*TMPRSS2-ERG* fusion occurs in 11% of the ductal component and in 5% of the acinar component of a mixed tumor, compared with 45%–50% of pure acinar adenocarcinomas [57, 58]. Similarly, *PTEN* is infrequently lost in the ductal and acinar components of mixed tumors and more often lost in pure acinar adenocarcinoma cells. These concordant characteristics of ductal and acinar components of mixed tumors suggest their clonal relationship. Gene expression profiling revealed remarkable similarity between ductal and typical acinar adenocarcinomas, with only 25 gene transcripts showing significant differences [59]. Transcripts significantly overexpressed in ductal adenocarcinomas include *CD24*, *CDH23* (cadherin-like 23), and *PRLR* (prolactin receptor). *CD24* and *CDH23* encode proteins with cell adhesion-related properties. *CD24* is a potential oncogene that is overexpressed in diverse malignancies, including prostatic cancer [60]. *PRLP* promotes ductal morphogenesis and is implicated in the development of the normal, hyperplastic, and neoplastic prostate. PRLP signaling contributes to tumorigenesis of the prostate and breast; therefore, targeting PRLP signaling attracts attention as a potential personalized therapy for prostatic cancer [61]. According to a recent study, somatic copy-number alterations (SCNAs) of ductal adenocarcinomas are similar to those of acinar adenocarcinomas of patients with a high GS (8–9) [62]. Chromosome 6q15 is frequently deleted in ductal adenocarcinoma cells, and deletions of *MAP3K7*, which is harbored in this locus, are associated with early biochemical recurrence, advanced tumor stage, and high GS. *MAP3K7* deletions are associated well with the *TMPRSS2-ERG* absence, which is more common in ductal than acinar adenocarcinomas [58, 63].

### Neuroendocrine carcinoma

LCNEC is newly adopted as a variant of neuroendocrine carcinoma, which comprises adenocarcinoma with neuroendocrine differentiation, small cell neuroendocrine carcinoma (or small cell carcinoma [SmCC]), and LCNEC.

Adenocarcinoma with neuroendocrine differentiation is defined as typical acinar or ductal adenocarcinoma without a detectable neuroendocrine morphology (such as nuclear molding and peripheral palisading) and with neuroendocrine differentiation, demonstrated only by immunohistochemical detection of at least one of three neuroendocrine markers (CHGA [chromogranin A], SYP [synaptophysin], or NCAM1 [CD56, NCAM]). Most studies did not detect an effect of neuroendocrine differentiation on clinical outcomes [1, 64]. Therefore, routine use of immunohistochemistry is not recommended to detect neuroendocrine differentiation in typical adenocarcinomas that lack neuroendocrine morphology.

Prostatic SmCC is extremely aggressive and is resistant to androgen-deprivation therapy (ADT). SmCC is characterized by scant cytoplasm (high nuclear to cytoplasmic ratio), hyperchromatic nuclei without conspicuous nucleoli, frequent mitosis, fragility, crush artifacts, nuclear molding, rosette-like structures, and geographic necrosis [1–3, 64]. Of importance, adenocarcinoma is initially diagnosed in one-third of patients with SmCC, followed by ADT, and subsequent diagnosis of SmCC. Approximately 50% of patients with SmCC present pure SmCC, whereas there is an admixture with acinar adenocarcinoma in the remaining patients. Immunohistochemically, prostatic SmCCs are positive for one or more of the three neuroendocrine markers and are infrequently positive for PSA and other prostatic markers. Immunohistochemistry reveals that SmCCs infrequently express TP63 (p63) and high-molecular weight cytokeratins, which are not detectably expressed by adenocarcinomas. Further, > 50% of prostatic SmCCs express NKX2-1 (TTF-1), limiting the utility of excluding a lung origin [1–3, 64].

Approximately 50% of prostatic SmCCs harbor *TMPRSS2-ERG*. Notably, unlike typical adenocarcinomas, such SmCCs do not consistently express ERG immunohistochemically, likely because of the lower frequency of expression of the androgen receptor (AR) by these cells [1–3, 64]. Further, an increased *AR* copy number may be associated with *TMPRSS2-ERG* in SmCCs. Inactivation of the tumor suppressors *RB1* and *TP53* commonly occurs in prostatic SmCCs, similar to their counterparts in the lung and other organs [65]. Inactivation of these genes through allelic loss or mutation, as well as lack of expression of their encoded proteins, is characteristic of prostatic SmCCs. The loss of RB1 expression occurs nearly universally in SmCCs and rarely occurs in high-grade acinar adenocarcinoma. Therefore, the loss of RB1 likely represents a critical event in the development of prostatic SmCC and may serve as a diagnostic marker and potential therapeutic target [65]. In xenograft models, prostatic SmCC is characterized by marked upregulation of *UBE2C* and other mitotic genes in the absence of *AR*, *RB1*, and *CCND1* (*cyclin D1*) expression [66].

Prostatic LCNEC is an aggressive tumor, with a mean survival of seven months, similar to SmCC [67]. LCNEC is extremely rare, particularly in its pure form. The largest series analyzed documents seven cases of LCNEC, among which only one case was a pure LCNEC and apparently de novo. Most cases arise in the setting of an existing acinar adenocarcinoma with a history of long-term ADT [67]. LCNEC is characterized by tumor cells with abundant cytoplasm, coarse nuclei with prominent nucleoli, and growth patterns of large nests, sheets, cords, and peripheral palisading. Diagnosing LCNEC requires observing these characteristic morphologies combined with immunohistochemical detection of at least one of the three neuroendocrine markers. LCNEC is negative or only focally positive for PSA using immunohistochemical detection. The Ki-67 index is typically > 50% and regional necrosis is commonly observed [1–3, 64]. Further accumulation of LCNEC cases is required to identify the clinicopathological and molecular hallmarks of this disease.

### Basal cell carcinoma (BCC)

BCC, which is extremely rare, resembles adenoid cystic carcinomas of the salivary glands. Microscopically, typical growth structures of BCC include adenoid cystic/cribriform patterns and small solid nests with palisading. BCC is typically characterized by hyperchromatic nuclei, a high nuclear to cytoplasmic ratio, lumens lined by eosinophilic cells, and desmoplastic or myxoid stroma. Immunohistochemical detection of BCL2 and a higher Ki-67 index favor a diagnosis of BCC vs basal cell hyperplasia [1, 2]. A subset of prostatic BCCs with adenoid cystic-like histology exhibits the *MYB* rearrangement, suggesting an independent entity [68].

### Squamous cell carcinoma (SqCC) and adenosquamous carcinoma

Squamous neoplasms comprise SqCC and adenosquamous carcinoma [1–3, 69]. Prostatic SqCC accounts for < 0.6% of prostatic cancers. Adenosquamous carcinomas occur less frequently. SqCC can originate in the periurethral glands or prostatic acini as well as from the lining basal cells. Patients with SqCC or adenosquamous carcinoma present with bladder-outlet obstruction and dysuria. The mean survival of patients with SqCC ranges from 6 to 24 months. Approximately 50% of SqCCs and adenosquamous carcinomas arise in patients with prostatic acinar adenocarcinoma, subsequent to ADT or radiotherapy, and may occur in association with prostatic schistosomiasis. SqCC often metastasizes to the bone, and the metastases are more frequently osteolytic than osteoblastic, which is observed in typical prostatic adenocarcinomas [1–3, 69].

The tumors are usually large, reaching 65 mm in the largest dimension. Prostatic SqCC must be distinguished from the involvement of SqCC of bladder origin and from squamous metaplasia occurring after ADT. Adenosquamous carcinoma is defined by the presence of SqCC and a glandular (acinar) adenocarcinoma component. The adenocarcinoma component is often of a high grade, whereas the grade of the SqCC component varies. Malignant squamous cells are often negative for PSA using immunohistochemistry [1–3, 69]. To my knowledge, molecular studies of prostatic SqCC or adenosquamous carcinoma are not published.

### Urothelial carcinoma

Prostatic urothelial carcinoma can arise from the urothelium of the prostatic urethra and the proximal portions of the prostatic ducts [1–3, 70]. Most cases of prostatic urothelial carcinoma present concurrently with bladder carcinoma. Patients present with hematuria, irritation, and obstructive symptoms. The clinical outcome of patients with prostatic urothelial carcinoma is usually dismal; however, the prognosis of patients with pure urothelial carcinoma *in situ* is excellent.

Prostatic urothelial carcinoma has a marked propensity for growth within prostatic ducts and acini with solid cylinders, with or without comedo necrosis [1–3, 70]. The tumor cells exhibit marked nuclear pleomorphism with numerous mitoses. Spreads between the basal cells and secretory cells in a single-cell pagetoid pattern are frequently observed. Stromal invasion is characterized by irregular nests and cords with a desmoplastic response. Immunohistochemically, tumors are positive for urothelial markers, including GATA3, and negative for prostate markers, including PSA and NKX3-1 (NKX3.1). There is no information, to my knowledge, indicating that the molecular features of this tumor differ from those of bladder urothelial carcinoma [1–3, 70].

### New variants of acinar adenocarcinoma of the prostate

The histological variants of acinar adenocarcinoma are updated in the 2016 WHO classification (Table 3). These variants are clinically important due to difficult diagnoses and prognostic differences, compared with typical acinar adenocarcinoma [3]. The histological variants are atrophic, pseudohyperplastic, microcystic, foamy gland, mucinous (colloid), signet ring-like cell, pleomorphic giant cell, and sarcomatoid. The newly recognized variants in the 2016 WHO classification are microcystic and pleomorphic giant cell [1]. Variants that are challenging to diagnose include benign-looking atrophic, pseudohyperplastic, microcystic, and foamy gland. The signet ring-like cell, pleomorphic giant cell, and sarcomatoid variants are associated with higher mortality than typical acinar adenocarcinomas.

**Table 3:** Variants of acinar adenocarcinoma (AC) of the prostate in the 2016 WHO classification [1]

Variants	Clinical Features
Atrophic	Resembling benign atrophic glands; cytoplasmic volume loss; GP 3
Pseudohyperplastic	Resembling benign luminal cell hyperplasia with papillary infoldings; GP 3; *HOXB3* G84E-related familial prostate cancer [36]
Microcystic	Benign-looking; dilated glands 10-times larger than glands of typical acinar AC; intraluminal crystalloids and wispy blue mucin; GP 3
Foamy gland	Benign-looking, abundant foamy cytoplasm; pyknotic nuclei
Mucinous (colloid)	At least 25% composed of extracellular mucin pools
Signet ring-like cell	Very aggressive; at least 25% composed of signet ring-like cells
Pleomorphic giant cell	Very aggressive; giant anaplastic cells with pleomorphic nuclei; lacking a spindle cell component
Sarcomatoid	Very aggressive; biphasic exhibiting epithelial and mesenchymal differentiations

AC, adenocarcinoma; GP, Gleason pattern; WHO, World Health Organization.

Sarcomatoid carcinoma, a variant of acinar adenocarcinoma, is also known as so-called carcinosarcoma. Sarcomatoid carcinoma represents a biphasic malignant tumor comprising adenocarcinoma and sarcomatoid components [1–3, 71]. Immunohistochemical analysis detects the expression of prostate markers, including PSA and NKX3-1, in cells of the adenocarcinoma component, whereas mesenchymal markers are detected in the sarcomatoid component. Approximately 50% of patients have a history of acinar adenocarcinoma treated with ADT, radiotherapy, or both. Therefore, the sarcomatoid component is likely to have evolved from the adenocarcinoma component. An LOH analysis found that adenocarcinoma and sarcomatoid components share a clonal origin [72]. A recent genomic assay using fluorescence in-situ hybridization found that the adenocarcinoma and sarcomatoid components harbor *ERG* fusions, indicating the epithelial origin of the sarcomatoid component [73].

Microcystic adenocarcinoma is a deceptively benign-appearing variant of acinar adenocarcinoma [74]. Microcystic glands are, on average, 10-times larger than glands of typical acinar adenocarcinoma. Intraluminal crystalloids and wispy blue intraluminal mucin are often detected. Atrophic features are observed focally, however the neoplastic lining cells exhibit a moderate amount of cytoplasm. Most microcystic adenocarcinomas possess alpha-methylacyl-CoA racemase (AMACR), and all of these tumors show evidence of complete basal cell loss, as determined by the immunohistochemical analysis of TP63 and 34βE12. The assigned GP is 3.

Pleomorphic giant cell adenocarcinoma, which is an extremely rare variant, comprises giant anaplastic cells with pleomorphic nuclei and lacks a spindle cell component [75, 76]. In addition to the pleomorphic giant cell element, a coexistent adenocarcinoma of Gleason score (GS) 9 is usually present. Evidence of focal ductal adenocarcinoma, squamous cell carcinoma, or SmCC is observed in some cases. The clinical course is typically very aggressive [1, 75, 76].

### Immunophenotype

For a diagnosis of prostate cancer, immunohistochemical analyses of expressions of PSA, prostatic acid phosphatase (PAP), high-molecular-weight cytokeratin (34βE12), TP63, and AMACR are helpful. The 2016 WHO classification introduces new immunohistochemical markers, including prostein (also known as P501S) and NKX3-1. Because PSA and PAP expression can decrease after ADT, NKX3-1 and prostein can be of use in such cases. Staining for prostein can be detected in cases of urinary bladder adenocarcinoma; however, its characteristic granular perinuclear pattern is not observed [77]. Nuclear NKX3-1 staining is highly specific for prostatic adenocarcinoma [78]. Prostatic markers of limited diagnostic utility, due to sensitivity and/or specificity, include prostate-specific membrane antigen (PSMA), ERG, AR, and AMACR.

**Figure 2:**
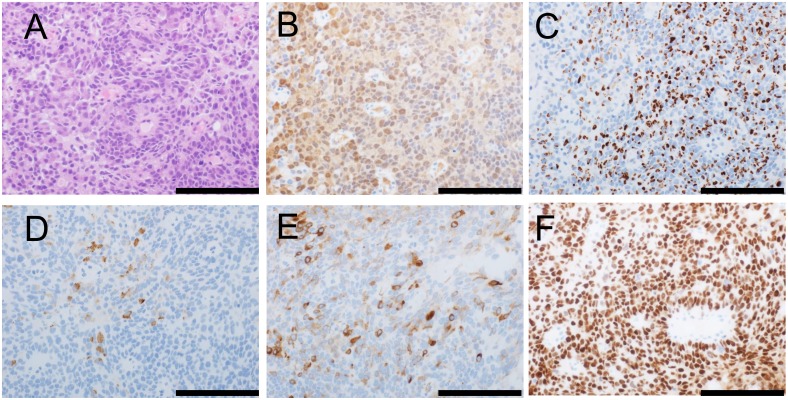
Morphology and immunophenotype of metastatic prostatic cancer in a neck lymph node Morphologically, the tumor is composed of round cells with solid growth (hematoxylin and eosin staining) (**A**). The tumor stained positive for NKX3-1 (**B**; nuclear staining), prostein (P501S) (**C**; granular perinuclear pattern), PSA (**D**), PSMA (**E**), and AR (**F**; nuclear staining). Scale bar, 100 μm.

An example of metastatic carcinoma of unknown origin that has spread to a neck lymph node is shown in Figure 2. Microscopically, the tumor is shown to be composed of round cells with solid growth (Figure 2A). The tumor stained positive for NKX3-1 (nuclear staining; Figure 2B), prostein (P501S) (granular perinuclear pattern; Figure 2C), PSA (Figure 2D), PSMA (Figure 2E), and AR (nuclear staining; Figure 2F). These immunohistochemical results support a diagnosis of metastatic prostatic cancer.

## MOLECULAR CHARACTERISTICS OF PROSTATIC CANCER

With the emergence of high-throughput sequencing techniques, detailed molecular profiles of prostatic cancer subtypes have been identified. The Cancer Genome Atlas (TCGA) research network identified genomic and other molecular alterations in different types of cancers, including prostatic cancers. The identification of subtypes with specific underlying genetic abnormalities can help identify potential therapeutic targets and predict effects on patient prognosis. In this section, the recently identified molecular characteristics of prostatic cancers are introduced.

### Comprehensive molecular subtyping by TCGA research network [5]

**Figure 3:**
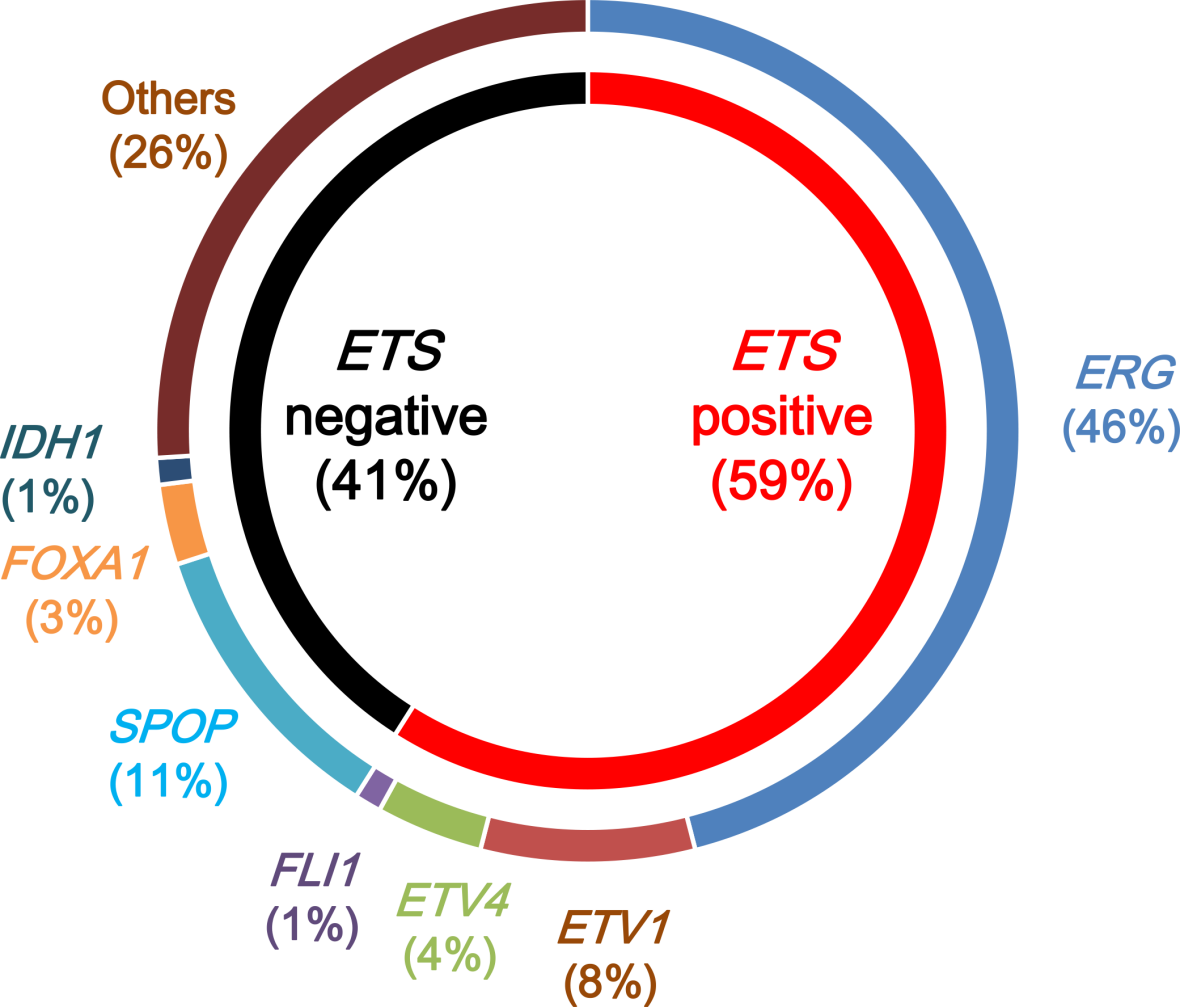
Molecular subtypes of primary prostatic cancers by The Cancer Genome Atlas Primary prostatic cancers can be classified into those with rearrangements in *ETS* family transcription factors (*ERG*, *ETV1*, *ETV4*, and *FLI1*) (*ETS* positive) and those without *ETS* rearrangements (*ETS* negative). *ETS*-positive prostatic cancers are classified by the specific *ETS*-fusion gene member involved: *ERG*, *ETV1*, *ETV4*, or *FLI1*. *ETS*-negative prostatic cancers are classified by mutations in *SPOP*, *FOXA1*, and *IDH1*.

The TCGA research network conducted a comprehensive molecular characterization of 333 primary prostatic cancers, including data on somatic mutations, gene fusions, SCNAs, gene expression, and DNA methylation [5]. Of these primary cancers, 75% were classified into seven subtypes, defined by either specific gene fusions of *ETS* transcription family members (*ERG*, *ETV1*, *ETV4*, and *FLI1*) or mutations (*SPOP*, *FOXA1*, and *IDH1*) (Figure 3). The *ETS*-positive subset, comprising 59% of all cases, was enriched in *PTEN* deletions. The *SPOP*-mutant subset, comprising 11% of all cases, harbored distinct SCNA profiles (including deletions of *CHD1*, 6q, and 2q), consistent with results from previous studies [14, 79]. The *SPOP*-mutant/*CHD1*-deleted subset harbored specific molecular characteristics, including high levels of DNA methylation, homogeneous gene expression patterns, and frequent overexpression of *SPINK1* mRNA. *SPOP*- and *FOXA1*-mutant subsets shared similar mRNA, SCNA, and DNA methylation profiles. Furthermore, a new and genomically distinct *IDH1*-mutant subset was identified. The mRNA clusters were tightly correlated with the *ETS*-fusion status: mRNA cluster 1 consisted of the *ETS*-negative subset, whereas mRNA clusters 2 and 3 both contained elements of the *ETS*-positive subset. MicroRNA results showed a similar pattern, indicating a general difference between the *ETS*-positive and -negative subsets. Clustering of expressed protein resulted in three distinct subgroups, with protein cluster 3 exhibiting elevated PIK3/AKT, MAP kinase, and receptor tyrosine kinase activities. However, this cluster was not enriched in genomic alterations of the analogous signaling pathways, and there were few associations of increased pathway activity.

The overall mutational burden was 0.94 mutations per megabase (median, range 0.04–24 per megabase), which corresponds to 19 nonsynonymous mutations per tumor genome (median, 13–25, 25th and 75th percentiles, respectively) [5]. This result is consistent with data from previous genome-scale sequencing studies of localized prostatic cancers [7, 14] and is lower than the mutational burden of metastatic prostatic cancers [8, 20, 21]. These findings confirmed that prostatic cancers carry a lower mutational burden than many other types of carcinoma that are not associated with a substantial exposure to a mutagen [80, 81]. The mutational significance analysis yielded 13 significantly mutated genes, including six previously identified mutations (*SPOP*, *TP53*, *FOXA1*, *PTEN*, *MED12*, and *CDKN1B*) and seven previously unidentified mutations (*BRAF*, *CTNNB1*, *HRAS*, *ATM*, *NKX3-1*, *AKT1*, and *ZMYM3*). Novel somatic mutations of *BRAF* (2.4%), *NKX3-1* (1%), and *ZMYM3* (2%) were included. Although the overall mutational burden was substantially higher in metastatic cancers, consistent with previous studies [20, 22], the primary and metastatic cancers were remarkably similar in their subtype distributions, except that metastatic cancers harbored no *IDH1* mutations.

Clustering results of the most variably hypermethylated CpG islands led to the identification of four epigenetically distinct groups. Nearly two-thirds of all *ERG*-rearranged cancers belonged to a cluster with only moderately elevated levels of methylation (DNA methylation cluster 3), whereas the remaining *ERG*-rearranged cancers comprised a distinct hypermethylated cluster (cluster 1) that was almost exclusively occupied by *ERG*-rearranged cancers. DNA methylation cluster 1 contained twice the number of hypermethylated loci in comparison with cluster 3, and the epigenetic patterns were substantially different from those of *ETV1*- and *ETV4*-rearranged cancers, which exhibit more heterogeneous methylation levels. This diversity among *ETS*-positive subtypes is consistent with results from previous studies that suggest substantial molecular and clinicopathological differences between *ERG*- and non-*ERG ETS*-rearranged cancers [82, 83]. *SPOP*- and *FOXA1*-mutant subsets showed homogeneous epigenetic profiles. These subsets belong almost exclusively to DNA methylation cluster 2, a cluster that also contains a majority of *ETV1*- and *ETV4*-, but not *ERG*-, rearranged subsets. Importantly, the *IDH1*-mutant subset harbors greater levels of genome-wide DNA hypermethylation. Integration of these epigenetic data with mRNA expression results yielded 164 genes that were epigenetically silenced in the subsets examined. *STAT6* was silenced predominantly in *ETS*-rearranged subsets, whereas *HEXA* was silenced frequently in the *SPOP*-mutant subset. As expected, the *IDH1*-mutant subset harbored the greatest number of epigenetically silenced genes among all prostatic cancers.

During assessments of AR activity, *ETS*-rearranged subsets showed variable AR transcriptional activity, whereas *SPOP*- and *FOXA1*-mutant subsets showed the highest AR transcriptional activity among all genotypically distinct subsets. Consistent with these findings, *SPOP* mutations were previously implicated in androgen signaling because both AR and AR coactivators undergo deregulation in the presence of *SPOP* mutations [84–86]. AR signaling was more frequently altered in metastatic samples, most often by amplifications or mutations of *AR*, which were essentially absent in localized prostatic cancers [87].

Several studies have indicated that DNA repair pathways are disrupted in a subgroup of prostatic cancers [38, 88, 89]. The poly ADP ribose polymerase (PARP) inhibitor olaparib is effective in some patients with prostate cancer [90]. Nearly 20% of all cases harbored the inactivation of DNA repair genes, including *BRCA2* (3%), *BRCA1* (1%), *CDK12* (2%), *ATM* (4%), *FANCD2* (7%), and *RAD51C* (3%) [5]. Recent evidence suggests that the incidence of germline mutations in genes mediating DNA repair processes was 11.8% in patients with metastatic prostatic cancer, which is significantly higher than in patients with localized prostatic cancer [38].

### *ETS* family

In 2005, genetic rearrangements of androgen-regulated genes with members of the *ETS* transcription factor family were identified in more than half of prostatic cancer cases [6, 10, 91, 92]. The most common rearrangement manifests as a fusion of the 5′ untranslated region of the androgen-regulated *TMPRSS2* gene with the coding area of *ERG*, accounting for 90% of *ETS* family fusions [92, 93]. Other members of the *ETS* family members include *ETV1*, *ETV4*, *ETV5*, and *FLI1* [93, 94]. *ETS* rearrangements have been occasionally detected in HGPIN and appear to be an early event in prostate carcinogenesis [95–97]. *ETS*-rearranged cancers are notably enriched in genomic alterations in a number of canonical pathways, including *PTEN* deletions, *TP53* alterations, PIK3 pathway alterations, and the specific amplification of 3p [98]. ERG overexpression accelerates prostatic carcinogenesis when combined with *PTEN* deletions [12, 13]. *PTEN* deletions are associated with metastatic disease, higher GP, higher risk of progression, recurrence after therapy, and death from diseases [99–104]. *ETS*-rearranged prostatic cancers exhibit characteristic SCNAs with a specific pattern of genomic rearrangements that involve chains of balanced alterations, a phenomenon known as “chromoplexy” [7, 18, 20, 105–107]. The *TMPRSS2-ERG* fusion increases bone tropism of prostatic cancers and promotes their metastases in bone [108]. Although the prognostic implications of *ETS* rearrangements remain unclear, recent evidence suggests that *TMPRSS2-ERG* fusions are associated with young patients and low-grade prostatic cancer [109]. A recent study demonstrates that small molecules targeting the DNA-binding ETS domain of *ERG* can suppress its transcriptional activity and reverse transformed characteristics of prostate cancers aberrantly expressing ERG [110].

### *SPOP* mutation

The *SPOP* mutation is the most common point mutation (6%–15%) in all prostatic cancers [14, 79, 111, 112]. The *SPOP* gene encodes the substrate-recognition component of the Cullin3-based E3-ubiquitin ligase; missense mutations are found exclusively in the substrate-binding cleft of *SPOP*, indicating that this mutation alters substrate binding [14]. *SPOP* mutations and *ETS* rearrangements are mutually exclusive. Because *SPOP* mutations can be detected in HGPINs adjacent to cancers, *SPOP* mutations are likely to be early events in prostatic carcinogenesis. Functionally, *SPOP* mutation promotes oncogenesis by pereventing the degradation of oncogenic facters including ERG and AR [84, 85, 113–115]. *SPOP*-mutant cancers generally lack genetic alterations in the PIK3 pathway [14, 20] but show a distinct pattern of genomic alterations, including losses of *CHD1* at 5q21, 2q, and 6q [14, 79]. CHD1 is an ATP-dependent chromatin-remodeling enzyme, whose genomic locus is lost in 5%–10% of all prostatic cancers [116, 117]. A recent study demonstrated that *CHD1* is a putative synthetic-essential gene in *PTEN*-deficient cancers, and *CHD1* depletion profoundly and specifically suppresses cell proliferation in *PTEN*-deficient prostate cancers [118]. Another recent study showed that *SPOP* mutations activate both PIK3/MTOR and AR signaling pathways, effectively uncoupling the normal negative-feedback mechanism between these two pathways [86].

### *SPINK1* overexpression

*SPINK1* is commonly overexpressed in *SPOP*-mutant and other *ETS*-negative prostatic cancers. *SPINK1* is overexpressed in approximately 10% of prostate cancers, and *SPINK1* overexpression and *ERG* rearrangement appear to be mutually exclusive. *SPINK1* overexpression is associated with an increased risk of biochemical recurrence [23]. Because *SPINK1* partially exhibits its neoplastic effects through its interaction with EGFR, EGFR inhibitors may be a potential targeted therapy for *SPINK1*-overexpressing prostatic cancers [119].

### *FOXA1* mutation

FOXA1 is an AR transcription factor that promotes prostatic cancer oncogenesis and progression mainly by increasing the transcriptional activity of AR [120]. *FOXA1*-mutant cancers share similar molecular features with *SPOP*-mutant cancers [5]. Along with *SPOP*-mutant cancers, *FOXA1*-mutant cancers have been associated with the highest levels of AR transcriptional activity [5]. A recent study suggested that NONOG, a pluripotency transcription factor, reprograms prostatic cancers to become castration resistant by dynamically repressing and engaging the AR/FOXA1 signaling axis [121]. Another recent study suggests that AR variants are dependent on FOXA1 for sustaining a pro-proliferative gene signatures and agents targeting FOXA1 may represent novel therapeutic options for patients with castrate-resistant prostatic cancer [122].

### *IDH1* mutation

*IDH1*, which encodes a metabolic enzyme, is recurrently mutated in prostatic cancers, resulting in a methylator phenotype [5]. The presence of *IDH1* mutations appears to represent a rare and unique subset of early onset prostate cancers, with relatively few SCNAs and high levels of genomic hypermethylation [5]. Patients with *IDH1*-mutant cancers might be candidates for treatment with IDH1-specific therapies that are currently under development [123–125].

## CONCLUSIONS AND FUTURE DIRECTIONS

This review provides perspectives on the new 2016 WHO classification of prostatic cancers as well as recent knowledge of their molecular pathologies. The comprehensive molecular characterization of prostatic cancers enabled their classification into subgroups, permitting their evolution from a poorly understood, heterogeneous group of diseases with variable clinical courses to characteristic molecular subtypes that could allow the implementation of personalized therapies and better patient management. In the clinical practice, although we have got to know the more precise prediction of prognosis or rough sensitivity for ADT by the histopathological and/or immunohistological examinations, the increased understanding of the molecular pathologies via high-throughput analyses are currently less useful for clinicians and pathologists to offer the meaningful therapeutic suggestions for patients with prostatic cancer. Actually, we continue to use mostly traditional approaches for the treatment of patients with prostatic cancer. Using the molecular knowledge of prostatic cancer, we really need to press forward the clinical translation and precision medicine for the patients. In accordance with the increased clinicopathological and molecular knowledge of prostatic cancers, the WHO classification will also require additional revisions to allow for reliable and clinically meaningful cancer diagnoses.

## Abbreviations

ADT: androgen deprivation therapy
AMACR: alpha-methylacyl-CoA racemase
AR: androgen receptor
BCC: basal cell carcinoma
CI: confidence interval
GP: Gleason pattern
GS: Gleason score
HGPIN: high-grade prostatic intraepithelial neoplasia
HR: hazard ratio
IDC-P: intraductal carcinoma of the prostate
ISUP: International Society of Urological Pathology
LCNEC: large cell neuroendocrine carcinoma
LOH: loss of heterozygosity
PAP: prostatic acid phosphatase
PARP: poly ADP ribose polymerase
PIN: prostatic intraepithelial neoplasia
PSA: prostate-specific antigen
PSMA: prostate-specific membrane antigen
SCNA: somatic copy-number alteration
SqCC: squamous cell carcinoma
SmCC: small cell carcinoma
TCGA: The Cancer Genome Atlas
WHO: World Health Organization

## References

[1] Moch H, Humphrey PA, Ulbright TM, Reuter VE (2016). WHO Classification of Tumours of the Urinary System and Male Genital Organs.

[2] Humphrey PA (2017). Histopathology of Prostate Cancer. Cold Spring Harb Perspect Med.

[3] Humphrey PA (2012). Histological variants of prostatic carcinoma and their significance. Histopathology.

[4] Humphrey PA, Moch H, Cubilla AL, Ulbright TM, Reuter VE (2016). The 2016 WHO Classification of Tumours of the Urinary System and Male Genital Organs-Part B: Prostate and Bladder Tumours. Eur Urol.

[5] Abeshouse A, Ahn J, Akbani R, Ally A, Amin S, Andry CD, Annala M, Aprikian A, Armenia J, Arora A, Auman JT, Balasundaram M, Balu S, Cancer Genome Atlas Research Network (2015). The Molecular Taxonomy of Primary Prostate Cancer. Cell.

[6] Kumar-Sinha C, Tomlins SA, Chinnaiyan AM (2008). Recurrent gene fusions in prostate cancer. Nat Rev Cancer.

[7] Baca SC, Prandi D, Lawrence MS, Mosquera JM, Romanel A, Drier Y, Park K, Kitabayashi N, MacDonald TY, Ghandi M, Van Allen E, Kryukov GV, Sboner A (2013). Punctuated evolution of prostate cancer genomes. Cell.

[8] Robinson D, Van Allen EM, Wu YM, Schultz N, Lonigro RJ, Mosquera JM, Montgomery B, Taplin ME, Pritchard CC, Attard G, Beltran H, Abida W, Bradley RK (2015). Integrative clinical genomics of advanced prostate cancer. Cell.

[9] Chang KH, Li R, Kuri B, Lotan Y, Roehrborn CG, Liu J, Vessella R, Nelson PS, Kapur P, Guo X, Mirzaei H, Auchus RJ, Sharifi N (2013). A gain-of-function mutation in DHT synthesis in castration-resistant prostate cancer. Cell.

[10] Tomlins SA, Rhodes DR, Perner S, Dhanasekaran SM, Mehra R, Sun XW, Varambally S, Cao X, Tchinda J, Kuefer R, Lee C, Montie JE, Shah RB (2005). Recurrent fusion of TMPRSS2 and ETS transcription factor genes in prostate cancer. Science.

[11] Xu K, Wu ZJ, Groner AC, He HH, Cai C, Lis RT, Wu X, Stack EC, Loda M, Liu T, Xu H, Cato L, Thornton JE (2012). EZH2 oncogenic activity in castration-resistant prostate cancer cells is Polycomb-independent. Science.

[12] King JC, Xu J, Wongvipat J, Hieronymus H, Carver BS, Leung DH, Taylor BS, Sander C, Cardiff RD, Couto SS, Gerald WL, Sawyers CL (2009). Cooperativity of TMPRSS2-ERG with PI3-kinase pathway activation in prostate oncogenesis. Nat Genet.

[13] Carver BS, Tran J, Gopalan A, Chen Z, Shaikh S, Carracedo A, Alimonti A, Nardella C, Varmeh S, Scardino PT, Cordon-Cardo C, Gerald W, Pandolfi PP (2009). Aberrant ERG expression cooperates with loss of PTEN to promote cancer progression in the prostate. Nat Genet.

[14] Barbieri CE, Baca SC, Lawrence MS, Demichelis F, Blattner M, Theurillat JP, White TA, Stojanov P, Van Allen E, Stransky N, Nickerson E, Chae SS, Boysen G (2012). Exome sequencing identifies recurrent SPOP, FOXA1 and MED12 mutations in prostate cancer. Nat Genet.

[15] Boutros PC, Fraser M, Harding NJ, de Borja R, Trudel D, Lalonde E, Meng A, Hennings-Yeomans PH, McPherson A, Sabelnykova VY, Zia A, Fox NS, Livingstone J (2015). Spatial genomic heterogeneity within localized, multifocal prostate cancer. Nat Genet.

[16] Kumar A, Coleman I, Morrissey C, Zhang X, True LD, Gulati R, Etzioni R, Bolouri H, Montgomery B, White T, Lucas JM, Brown LG, Dumpit RF (2016). Substantial interindividual and limited intraindividual genomic diversity among tumors from men with metastatic prostate cancer. Nat Med.

[17] Varambally S, Dhanasekaran SM, Zhou M, Barrette TR, Kumar-Sinha C, Sanda MG, Ghosh D, Pienta KJ, Sewalt RG, Otte AP, Rubin MA, Chinnaiyan AM (2002). The polycomb group protein EZH2 is involved in progression of prostate cancer. Nature.

[18] Tomlins SA, Laxman B, Dhanasekaran SM, Helgeson BE, Cao X, Morris DS, Menon A, Jing X, Cao Q, Han B, Yu J, Wang L, Montie JE (2007). Distinct classes of chromosomal rearrangements create oncogenic ETS gene fusions in prostate cancer. Nature.

[19] Berger MF, Lawrence MS, Demichelis F, Drier Y, Cibulskis K, Sivachenko AY, Sboner A, Esgueva R, Pflueger D, Sougnez C, Onofrio R, Carter SL, Park K (2011). The genomic complexity of primary human prostate cancer. Nature.

[20] Grasso CS, Wu YM, Robinson DR, Cao X, Dhanasekaran SM, Khan AP, Quist MJ, Jing X, Lonigro RJ, Brenner JC, Asangani IA, Ateeq B, Chun SY (2012). The mutational landscape of lethal castration-resistant prostate cancer. Nature.

[21] Gundem G, Van Loo P, Kremeyer B, Alexandrov LB, Tubio JM, Papaemmanuil E, Brewer DS, Kallio HM, Högnäs G, Annala M, Kivinummi K, Goody V, Latimer C, ICGC Prostate Group (2015). The evolutionary history of lethal metastatic prostate cancer. Nature.

[22] Taylor BS, Schultz N, Hieronymus H, Gopalan A, Xiao Y, Carver BS, Arora VK, Kaushik P, Cerami E, Reva B, Antipin Y, Mitsiades N, Landers T (2010). Integrative genomic profiling of human prostate cancer. Cancer Cell.

[23] Tomlins SA, Rhodes DR, Yu J, Varambally S, Mehra R, Perner S, Demichelis F, Helgeson BE, Laxman B, Morris DS, Cao Q, Cao X, Andren O (2008). The role of SPINK1 in ETS rearrangement-negative prostate cancers. Cancer Cell.

[24] Shen MM (2013). Chromoplexy: a new category of complex rearrangements in the cancer genome. Cancer Cell.

[25] Brenner JC, Ateeq B, Li Y, Yocum AK, Cao Q, Asangani IA, Patel S, Wang X, Liang H, Yu J, Palanisamy N, Siddiqui J, Yan W (2011). Mechanistic rationale for inhibition of poly(ADP-ribose) polymerase in ETS gene fusion-positive prostate cancer. Cancer Cell.

[26] Chen M, Pratt CP, Zeeman ME, Schultz N, Taylor BS, O’Neill A, Castillo-Martin M, Nowak DG, Naguib A, Grace DM, Murn J, Navin N, Atwal GS (2011). Identification of PHLPP1 as a tumor suppressor reveals the role of feedback activation in PTEN-mutant prostate cancer progression. Cancer Cell.

[27] Weischenfeldt J, Simon R, Feuerbach L, Schlangen K, Weichenhan D, Minner S, Wuttig D, Warnatz HJ, Stehr H, Rausch T, Jager N, Gu L, Bogatyrova O (2013). Integrative genomic analyses reveal an androgen-driven somatic alteration landscape in early-onset prostate cancer. Cancer Cell.

[28] Bertoli G, Cava C, Castiglioni I (2016). MicroRNAs as Biomarkers for Diagnosis, Prognosis and Theranostics in Prostate Cancer. Int J Mol Sci.

[29] Takayama KI, Misawa A, Inoue S (2017). Significance of microRNAs in Androgen Signaling and Prostate Cancer Progression. Cancers (Basel).

[30] Crumbaker M, Khoja L, Joshua AM (2017). AR Signaling and the PI3K Pathway in Prostate Cancer. Cancers (Basel).

[31] Eisermann K, Fraizer G (2017). The Androgen Receptor and VEGF: Mechanisms of Androgen-Regulated Angiogenesis in Prostate Cancer. Cancers (Basel).

[32] Obinata D, Takayama K, Takahashi S, Inoue S (2017). Crosstalk of the Androgen Receptor with Transcriptional Collaborators: Potential Therapeutic Targets for Castration-Resistant Prostate Cancer. Cancers (Basel).

[33] Pakula H, Xiang D, Li Z (2017). A Tale of Two Signals: AR and WNT in Development and Tumorigenesis of Prostate and Mammary Gland. Cancers (Basel).

[34] Cucchiara V, Yang JC, Mirone V, Gao AC, Rosenfeld MG, Evans CP (2017). Epigenomic Regulation of Androgen Receptor Signaling: Potential Role in Prostate Cancer Therapy. Cancers (Basel).

[35] Barbieri CE, Tomlins SA (2014). The prostate cancer genome: perspectives and potential. Urol Oncol.

[36] Smith SC, Palanisamy N, Zuhlke KA, Johnson AM, Siddiqui J, Chinnaiyan AM, Kunju LP, Cooney KA, Tomlins SA (2014). HOXB13 G84E-related familial prostate cancers: a clinical, histologic, and molecular survey. Am J Surg Pathol.

[37] Beltran H, Yelensky R, Frampton GM, Park K, Downing SR, MacDonald TY, Jarosz M, Lipson D, Tagawa ST, Nanus DM, Stephens PJ, Mosquera JM, Cronin MT (2013). Targeted next-generation sequencing of advanced prostate cancer identifies potential therapeutic targets and disease heterogeneity. Eur Urol.

[38] Pritchard CC, Mateo J, Walsh MF, De Sarkar N, Abida W, Beltran H, Garofalo A, Gulati R, Carreira S, Eeles R, Elemento O, Rubin MA, Robinson D (2016). Inherited DNA-Repair Gene Mutations in Men with Metastatic Prostate Cancer. N Engl J Med.

[39] Zhao S, Løvf M, Carm KT, Bakken AC, Hoff AM, Skotheim RI (2017). Novel transcription-induced fusion RNAs in prostate cancer. Oncotarget.

[40] Vanacore D, Boccellino M, Rossetti S, Cavaliere C, D’Aniello C, Di Franco R, Romano FJ, Montanari M, La Mantia E, Piscitelli R, Nocerino F, Cappuccio F, Grimaldi G (2017). Micrornas in prostate cancer: an overview. Oncotarget.

[41] Jhun MA, Geybels MS, Wright JL, Kolb S, April C, Bibikova M, Ostrander EA, Fan JB, Feng Z, Stanford JL (2017). Gene expression signature of Gleason score is associated with prostate cancer outcomes in a radical prostatectomy cohort. Oncotarget.

[42] Montanari M, Rossetti S, Cavaliere C, D’Aniello C, Malzone MG, Vanacore D, Di Franco R, La Mantia E, Iovane G, Piscitelli R, Muscariello R, Berretta M, Perdona S (2017). Epithelial-mesenchymal transition in prostate cancer: an overview. Oncotarget.

[43] Lin D, Ettinger SL, Qu S, Xue H, Nabavi N, Choi SYC, Bell RH, Mo F, Haegert AM, Gout PW, Fleshner N, Gleave ME, Pollak M (2017). Metabolic heterogeneity signature of primary treatment-naive prostate cancer. Oncotarget.

[44] Eble JN, Sauter G, Epstein JI, Sesterhenn IA (2004). World Health Organization Classification of Tumours. Pathology and Genetics of Tumours of the Urinary System and Male Genital Organs.

[45] Epstein JI, Egevad L, Amin MB, Delahunt B, Srigley JR, Humphrey PA (2016). The 2014 International Society of Urological Pathology (ISUP) Consensus Conference on Gleason Grading of Prostatic Carcinoma: Definition of Grading Patterns and Proposal for a New Grading System. Am J Surg Pathol.

[46] Pierorazio PM, Walsh PC, Partin AW, Epstein JI (2013). Prognostic Gleason grade grouping: data based on the modified Gleason scoring system. BJU Int.

[47] Epstein JI, Zelefsky MJ, Sjoberg DD, Nelson JB, Egevad L, Magi-Galluzzi C, Vickers AJ, Parwani AV, Reuter VE, Fine SW, Eastham JA, Wiklund P, Han M (2016). A Contemporary Prostate Cancer Grading System: A Validated Alternative to the Gleason Score. Eur Urol.

[48] Ham WS, Chalfin HJ, Feng Z, Trock BJ, Epstein JI, Cheung C, Humphreys E, Partin AW, Han M (2017). New Prostate Cancer Grading System Predicts Long-term Survival Following Surgery for Gleason Score 8-10 Prostate Cancer. Eur Urol.

[49] Guo CC, Epstein JI (2006). Intraductal carcinoma of the prostate on needle biopsy: Histologic features and clinical significance. Mod Pathol.

[50] Robinson BD, Epstein JI (2010). Intraductal carcinoma of the prostate without invasive carcinoma on needle biopsy: emphasis on radical prostatectomy findings. J Urol.

[51] Watts K, Li J, Magi-Galluzzi C, Zhou M (2013). Incidence and clinicopathological characteristics of intraductal carcinoma detected in prostate biopsies: a prospective cohort study. Histopathology.

[52] Dawkins HJ, Sellner LN, Turbett GR, Thompson CA, Redmond SL, McNeal JE, Cohen RJ (2000). Distinction between intraductal carcinoma of the prostate (IDC-P), high-grade dysplasia (PIN), and invasive prostatic adenocarcinoma, using molecular markers of cancer progression. Prostate.

[53] Han B, Suleman K, Wang L, Siddiqui J, Sercia L, Magi-Galluzzi C, Palanisamy N, Chinnaiyan AM, Zhou M, Shah RB (2010). ETS gene aberrations in atypical cribriform lesions of the prostate: Implications for the distinction between intraductal carcinoma of the prostate and cribriform high-grade prostatic intraepithelial neoplasia. Am J Surg Pathol.

[54] Lotan TL, Gumuskaya B, Rahimi H, Hicks JL, Iwata T, Robinson BD, Epstein JI, De Marzo AM (2013). Cytoplasmic PTEN protein loss distinguishes intraductal carcinoma of the prostate from high-grade prostatic intraepithelial neoplasia. Mod Pathol.

[55] Morgan TM, Welty CJ, Vakar-Lopez F, Lin DW, Wright JL (2010). Ductal adenocarcinoma of the prostate: increased mortality risk and decreased serum prostate specific antigen. J Urol.

[56] Ellis CL, Epstein JI (2015). Metastatic prostate adenocarcinoma to the penis: a series of 29 cases with predilection for ductal adenocarcinoma. Am J Surg Pathol.

[57] Lotan TL, Toubaji A, Albadine R, Latour M, Herawi M, Meeker AK, DeMarzo AM, Platz EA, Epstein JI, Netto GJ (2009). TMPRSS2-ERG gene fusions are infrequent in prostatic ductal adenocarcinomas. Mod Pathol.

[58] Morais CL, Herawi M, Toubaji A, Albadine R, Hicks J, Netto GJ, De Marzo AM, Epstein JI, Lotan TL (2015). PTEN loss and ERG protein expression are infrequent in prostatic ductal adenocarcinomas and concurrent acinar carcinomas. Prostate.

[59] Sanati S, Watson MA, Salavaggione AL, Humphrey PA (2009). Gene expression profiles of ductal versus acinar adenocarcinoma of the prostate. Mod Pathol.

[60] Kristiansen G, Sammar M, Altevogt P (2004). Tumour biological aspects of CD24, a mucin-like adhesion molecule. J Mol Histol.

[61] Goffin V (2017). Prolactin receptor targeting in breast and prostate cancers: New insights into an old challenge. Pharmacol Ther.

[62] Seipel AH, Whitington T, Delahunt B, Samaratunga H, Mayrhofer M, Wiklund P, Grönberg H, Lindberg J, Egevad L (2017). Genetic profile of ductal adenocarcinoma of the prostate. Hum Pathol.

[63] Kluth M, Hesse J, Heinl A, Krohn A, Steurer S, Sirma H, Simon R, Mayer PS, Schumacher U, Grupp K, Izbicki JR, Pantel K, Dikomey E (2013). Genomic deletion of MAP3K7 at 6q12-22 is associated with early PSA recurrence in prostate cancer and absence of TMPRSS2: ERG fusions. Mod Pathol.

[64] Epstein JI, Amin MB, Beltran H, Lotan TL, Mosquera JM, Reuter VE, Robinson BD, Troncoso P, Rubin MA (2014). Proposed morphologic classification of prostate cancer with neuroendocrine differentiation. Am J Surg Pathol.

[65] Tan HL, Sood A, Rahimi HA, Wang W, Gupta N, Hicks J, Mosier S, Gocke CD, Epstein JI, Netto GJ, Liu W, Isaacs WB, De Marzo AM (2014). Rb loss is characteristic of prostatic small cell neuroendocrine carcinoma. Clin Cancer Res.

[66] Tzelepi V, Zhang J, Lu JF, Kleb B, Wu G, Wan X, Hoang A, Efstathiou E, Sircar K, Navone NM, Troncoso P, Liang S, Logothetis CJ (2012). Modeling a lethal prostate cancer variant with small-cell carcinoma features. Clin Cancer Res.

[67] Evans AJ, Humphrey PA, Belani J, van der Kwast TH, Srigley JR (2006). Large cell neuroendocrine carcinoma of prostate: a clinicopathologic summary of 7 cases of a rare manifestation of advanced prostate cancer. Am J Surg Pathol.

[68] Bishop JA, Yonescu R, Epstein JI, Westra WH (2015). A subset of prostatic basal cell carcinomas harbor the MYB rearrangement of adenoid cystic carcinoma. Hum Pathol.

[69] Parwani AV, Kronz JD, Genega EM, Gaudin P, Chang S, Epstein JI (2004). Prostate carcinoma with squamous differentiation: an analysis of 33 cases. Am J Surg Pathol.

[70] Cheville JC, Dundore PA, Bostwick DG, Lieber MM, Batts KP, Sebo TJ, Farrow GM (1998). Transitional cell carcinoma of the prostate: clinicopathologic study of 50 cases. Cancer.

[71] Markowski MC, Eisenberger MA, Zahurak M, Epstein JI, Paller CJ (2015). Sarcomatoid Carcinoma of the Prostate: Retrospective Review of a Case Series From the Johns Hopkins Hospital. Urology.

[72] Ray ME, Wojno KJ, Goldstein NS, Olson KB, Shah RB, Cooney KA (2006). Clonality of sarcomatous and carcinomatous elements in sarcomatoid carcinoma of the prostate. Urology.

[73] Rodrigues DN, Hazell S, Miranda S, Crespo M, Fisher C, de Bono JS, Attard G (2015). Sarcomatoid carcinoma of the prostate: ERG fluorescence in-situ hybridization confirms epithelial origin. Histopathology.

[74] Yaskiv O, Cao D, Humphrey PA (2010). Microcystic adenocarcinoma of the prostate: a variant of pseudohyperplastic and atrophic patterns. Am J Surg Pathol.

[75] Lopez-Beltran A, Eble JN, Bostwick DG (2005). Pleomorphic giant cell carcinoma of the prostate. Arch Pathol Lab Med.

[76] Parwani AV, Herawi M, Epstein JI (2006). Pleomorphic giant cell adenocarcinoma of the prostate: report of 6 cases. Am J Surg Pathol.

[77] Lane Z, Hansel DE, Epstein JI (2008). Immunohistochemical expression of prostatic antigens in adenocarcinoma and villous adenoma of the urinary bladder. Am J Surg Pathol.

[78] Gurel B, Ali TZ, Montgomery EA, Begum S, Hicks J, Goggins M, Eberhart CG, Clark DP, Bieberich CJ, Epstein JI, De Marzo AM (2010). NKX3.1 as a marker of prostatic origin in metastatic tumors. Am J Surg Pathol.

[79] Blattner M, Lee DJ, O’Reilly C, Park K, MacDonald TY, Khani F, Turner KR, Chiu YL, Wild PJ, Dolgalev I, Heguy A, Sboner A, Ramazangolu S (2014). SPOP mutations in prostate cancer across demographically diverse patient cohorts. Neoplasia.

[80] Alexandrov LB, Nik-Zainal S, Wedge DC, Aparicio SA, Behjati S, Biankin AV, Bignell GR, Bolli N, Borg A, Børresen-Dale AL, Boyault S, Burkhardt B, Butler AP, Australian Pancreatic Cancer Genome Initiative, and ICGC Breast Cancer Consortium, and ICGC MMML- Seq Consortium, and ICGC PedBrain (2013). Signatures of mutational processes in human cancer. Nature.

[81] Lawrence MS, Stojanov P, Polak P, Kryukov GV, Cibulskis K, Sivachenko A, Carter SL, Stewart C, Mermel CH, Roberts SA, Kiezun A, Hammerman PS, McKenna A (2013). Mutational heterogeneity in cancer and the search for new cancer-associated genes. Nature.

[82] Baena E, Shao Z, Linn DE, Glass K, Hamblen MJ, Fujiwara Y, Kim J, Nguyen M, Zhang X, Godinho FJ, Bronson RT, Mucci LA, Loda M (2013). ETV1 directs androgen metabolism and confers aggressive prostate cancer in targeted mice and patients. Genes Dev.

[83] Tomlins SA, Alshalalfa M, Davicioni E, Erho N, Yousefi K, Zhao S, Haddad Z, Den RB Dicker AP, Trock BJ, DeMarzo AM, Ross AE, Schaeffer EM (2015). Characterization of 1577 primary prostate cancers reveals novel biological and clinicopathologic insights into molecular subtypes. Eur Urol.

[84] An J, Wang C, Deng Y, Yu L, Huang H (2014). Destruction of full-length androgen receptor by wild-type SPOP, but not prostate-cancer-associated mutants. Cell Rep.

[85] Geng C, Rajapakshe K, Shah SS, Shou J, Eedunuri VK, Foley C, Fiskus W, Rajendran M, Chew SA, Zimmermann M, Bond R, He B, Coarfa C (2014). Androgen receptor is the key transcriptional mediator of the tumor suppressor SPOP in prostate cancer. Cancer Res.

[86] Blattner M, Liu D, Robinson BD, Huang D, Poliakov A, Gao D, Nataraj S, Deonarine LD, Augello MA, Sailer V, Ponnala L, Ittmann M, Chinnaiyan AM (2017). SPOP Mutation Drives Prostate Tumorigenesis *In Vivo* through Coordinate Regulation of PI3K/mTOR and AR Signaling. Cancer Cell.

[87] Galletti G, Leach BI, Lam L, Tagawa ST (2017). Mechanisms of resistance to systemic therapy in metastatic castration-resistant prostate cancer. Cancer Treat Rev.

[88] Karanika S, Karantanos T, Li L, Corn PG, Thompson TC (2015). DNA damage response and prostate cancer: defects, regulation and therapeutic implications. Oncogene.

[89] Pritchard CC, Morrissey C, Kumar A, Zhang X, Smith C, Coleman I, Salipante SJ, Milbank J, Yu M, Grady WM, Tait JF, Corey E, Vessella RL (2014). Complex MSH2 and MSH6 mutations in hypermutated microsatellite unstable advanced prostate cancer. Nat Commun.

[90] Rimar KJ, Tran PT, Matulewicz RS, Hussain M, Meeks JJ (2017). The emerging role of homologous recombination repair and PARP inhibitors in genitourinary malignancies. Cancer.

[91] Tomlins SA, Mehra R, Rhodes DR, Smith LR, Roulston D, Helgeson BE, Cao X, Wei JT, Rubin MA, Shah RB, Chinnaiyan AM (2006). TMPRSS2: ETV4 gene fusions define a third molecular subtype of prostate cancer. Cancer Res.

[92] Perner S, Demichelis F, Beroukhim R, Schmidt FH, Mosquera JM, Setlur S, Tchinda J, Tomlins SA, Hofer MD, Pienta KG, Kuefer R, Vessella R, Sun XW (2006). TMPRSS2: ERG fusion-associated deletions provide insight into the heterogeneity of prostate cancer. Cancer Res.

[93] Tomlins SA, Bjartell A, Chinnaiyan AM, Jenster G, Nam RK, Rubin MA, Schalken JA (2009). ETS gene fusions in prostate cancer: from discovery to daily clinical practice. Eur Urol.

[94] Paulo P, Barros-Silva JD, Ribeiro FR, Ramalho-Carvalho J, Jeronimo C, Henrique R, Lind GE, Skotheim RI, Lothe RA, Teixeira MR (2012). FLI1 is a novel ETS transcription factor involved in gene fusions in prostate cancer. Genes Chromosomes Cancer.

[95] Perner S, Mosquera JM, Demichelis F, Hofer MD, Paris PL, Simko J, Collins C, Bismar TA, Chinnaiyan AM, De Marzo AM, Rubin MA (2007). TMPRSS2-ERG fusion prostate cancer: an early molecular event associated with invasion. Am J Surg Pathol.

[96] Park K, Tomlins SA, Mudaliar KM, Chiu YL, Esgueva R, Mehra R, Suleman K, Varambally S, Brenner JC, MacDonald T, Srivastava A, Tewari AK, Sathyanarayana U (2010). Antibody-based detection of ERG rearrangement-positive prostate cancer. Neoplasia.

[97] Morais CL, Guedes LB, Hicks J, Baras AS, De Marzo AM, Lotan TL (2016). ERG and PTEN status of isolated high-grade PIN occurring in cystoprostatectomy specimens without invasive prostatic adenocarcinoma. Hum Pathol.

[98] Kaffenberger SD, Barbieri CE (2016). Molecular subtyping of prostate cancer. Curr Opin Urol.

[99] Attard G, Swennenhuis JF, Olmos D, Reid AH, Vickers E, A’Hern R, Levink R, Coumans F, Moreira J, Riisnaes R, Oommen NB, Hawche G, Jameson C (2009). Characterization of ERG, AR and PTEN gene status in circulating tumor cells from patients with castration-resistant prostate cancer. Cancer Res.

[100] Choucair K, Ejdelman J, Brimo F, Aprikian A, Chevalier S, Lapointe J (2012). PTEN genomic deletion predicts prostate cancer recurrence and is associated with low AR expression and transcriptional activity. BMC Cancer.

[101] McMenamin ME, Soung P, Perera S, Kaplan I, Loda M, Sellers WR (1999). Loss of PTEN expression in paraffin-embedded primary prostate cancer correlates with high Gleason score and advanced stage. Cancer Res.

[102] Reid AH, Attard G, Ambroisine L, Fisher G, Kovacs G, Brewer D, Clark J, Flohr P, Edwards S, Berney DM, Foster CS, Fletcher A, Gerald WL (2010). Molecular characterisation of ERG, ETV1 and PTEN gene loci identifies patients at low and high risk of death from prostate cancer. Br J Cancer.

[103] Cairns P, Okami K, Halachmi S, Halachmi N, Esteller M, Herman JG, Jen J, Isaacs WB, Bova GS, Sidransky D (1997). Frequent inactivation of PTEN/MMAC1 in primary prostate cancer. Cancer Res.

[104] Krohn A, Diedler T, Burkhardt L, Mayer PS, De Silva C, Meyer-Kornblum M, Kotschau D, Tennstedt P, Huang J, Gerhauser C, Mader M, Kurtz S, Sirma H (2012). Genomic deletion of PTEN is associated with tumor progression and early PSA recurrence in ERG fusion-positive and fusion-negative prostate cancer. Am J Pathol.

[105] Metzger E, Willmann D, McMillan J, Forne I, Metzger P, Gerhardt S, Petroll K, von Maessenhausen A, Urban S, Schott AK, Espejo A, Eberlin A, Wohlwend D (2016). Assembly of methylated KDM1A and CHD1 drives androgen receptor-dependent transcription and translocation. Nat Struct Mol Biol.

[106] Setlur SR, Mertz KD, Hoshida Y, Demichelis F, Lupien M, Perner S, Sboner A, Pawitan Y, Andren O, Johnson LA, Tang J, Adami HO, Calza S (2008). Estrogen-dependent signaling in a molecularly distinct subclass of aggressive prostate cancer. J Natl Cancer Inst.

[107] Demichelis F, Rubin MA (2007). TMPRSS2-ETS fusion prostate cancer: biological and clinical implications. J Clin Pathol.

[108] Deplus R, Delliaux C, Marchand N, Flourens A, Vanpouille N, Leroy X, de Launoit Y, Duterque-Coquillaud M (2017). TMPRSS2-ERG fusion promotes prostate cancer metastases in bone. Oncotarget.

[109] Steurer S, Mayer PS, Adam M, Krohn A, Koop C, Ospina-Klinck D, Tehrani AA, Simon R, Tennstedt P, Graefen M, Wittmer C, Brors B, Plass C (2014). TMPRSS2-ERG fusions are strongly linked to young patient age in low-grade prostate cancer. Eur Urol.

[110] Butler MS, Roshan-Moniri M, Hsing M, Lau D, Kim A, Yen P, Mroczek M, Nouri M, Lien S, Axerio-Cilies P, Dalal K, Yau C, Ghaidi F (2017). Discovery and characterization of small molecules targeting the DNA-binding ETS domain of ERG in prostate cancer. Oncotarget.

[111] Lindberg J, Klevebring D, Liu W, Neiman M, Xu J, Wiklund P, Wiklund F, Mills IG, Egevad L, Gronberg H (2013). Exome sequencing of prostate cancer supports the hypothesis of independent tumour origins. Eur Urol.

[112] Lindberg J, Mills IG, Klevebring D, Liu W, Neiman M, Xu J, Wikstrom P, Wiklund P, Wiklund F, Egevad L, Gronberg H (2013). The mitochondrial and autosomal mutation landscapes of prostate cancer. Eur Urol.

[113] Theurillat JP, Udeshi ND, Errington WJ, Svinkina T, Baca SC, Pop M, Wild PJ, Blattner M, Groner AC, Rubin MA, Moch H, Prive GG, Carr SA, Prostate cancer (2014). Ubiquitylome analysis identifies dysregulation of effector substrates in SPOP-mutant prostate cancer. Science.

[114] An J, Ren S, Murphy SJ, Dalangood S, Chang C, Pang X, Cui Y, Wang L, Pan Y, Zhang X, Zhu Y, Wang C, Halling GC (2015). Truncated ERG Oncoproteins from TMPRSS2-ERG Fusions Are Resistant to SPOP-Mediated Proteasome Degradation. Mol Cell.

[115] Gan W, Dai X, Lunardi A, Li Z, Inuzuka H, Liu P, Varmeh S, Zhang J, Cheng L, Sun Y, Asara JM, Beck AH, Huang J (2015). SPOP Promotes Ubiquitination and Degradation of the ERG Oncoprotein to Suppress Prostate Cancer Progression. Mol Cell.

[116] Liu W, Lindberg J, Sui G, Luo J, Egevad L, Li T, Xie C, Wan M, Kim ST, Wang Z, Turner AR, Zhang Z, Feng J (2012). Identification of novel CHD1-associated collaborative alterations of genomic structure and functional assessment of CHD1 in prostate cancer. Oncogene.

[117] Huang S, Gulzar ZG, Salari K, Lapointe J, Brooks JD, Pollack JR (2012). Recurrent deletion of CHD1 in prostate cancer with relevance to cell invasiveness. Oncogene.

[118] Zhao D, Lu X, Wang G, Lan Z, Liao W, Li J, Liang X, Chen JR, Shah S, Shang X, Tang M, Deng P, Dey P (2017). Synthetic essentiality of chromatin remodelling factor CHD1 in PTEN-deficient cancer. Nature.

[119] Ateeq B, Tomlins SA, Laxman B, Asangani IA, Cao Q, Cao X, Li Y, Wang X, Feng FY, Pienta KJ, Varambally S, Chinnaiyan AM (2011). Therapeutic targeting of SPINK1-positive prostate cancer. Sci Transl Med.

[120] Jin HJ, Zhao JC, Ogden I, Bergan RC, Yu J (2013). Androgen receptor-independent function of FoxA1 in prostate cancer metastasis. Cancer Res.

[121] Jeter CR, Liu B, Lu Y, Chao HP, Zhang D, Liu X, Chen X, Li Q, Rycaj K, Calhoun-Davis T, Yan L, Hu Q, Wang J (2016). NANOG reprograms prostate cancer cells to castration resistance via dynamically repressing and engaging the AR/FOXA1 signaling axis. Cell Discov.

[122] Jones D, Wade M, Nakjang S, Chaytor L, Grey J, Robson CN, Gaughan L (2015). FOXA1 regulates androgen receptor variant activity in models of castrate-resistant prostate cancer. Oncotarget.

[123] Rohle D, Popovici-Muller J, Palaskas N, Turcan S, Grommes C, Campos C, Tsoi J, Clark O, Oldrini B, Komisopoulou E, Kunii K, Pedraza A, Schalm S (2013). An inhibitor of mutant IDH1 delays growth and promotes differentiation of glioma cells. Science.

[124] Schumacher T, Bunse L, Pusch S, Sahm F, Wiestler B, Quandt J, Menn O, Osswald M, Oezen I, Ott M, Keil M, Balss J, Rauschenbach K (2014). A vaccine targeting mutant IDH1 induces antitumour immunity. Nature.

[125] Spratt DE, Zumsteg ZS, Feng FY, Tomlins SA (2016). Translational and clinical implications of the genetic landscape of prostate cancer. Nat Rev Clin Oncol.

